# Baseline Characteristics and Risk Factors of Pulmonary Arterial Hypertension in Systemic Lupus Erythematosus Patients

**DOI:** 10.1097/MD.0000000000002761

**Published:** 2016-03-11

**Authors:** Can Huang, Mengtao Li, Yongtai Liu, Qian Wang, Xiaoxiao Guo, Jiuliang Zhao, Jinzhi Lai, Zhuang Tian, Yan Zhao, Xiaofeng Zeng

**Affiliations:** From the Department of Rheumatology (CH, ML, QW, JZ, YZ, XZ), Peking Union Medical College Hospital, Key Laboratory of Rheumatology and Clinical Immunology, Ministry of Education and Department of Cardiology (YL, XG, JL, ZT), Peking Union Medical College Hospital, Chinese Academy of Medical Science and Peking Union Medical College, Beijing, China.

## Abstract

Peking Union Medical College Hospital (PUMCH) has started a single-center right heart catheterization (RHC)-based pulmonary arterial hypertension (PAH) study in systemic lupus erythematosus (SLE) since 2006. The baseline characteristics of these patients were described and the risk factor for PAH in lupus was identified.

The demographic, clinical, laboratory, and treatment characteristics of SLE patients with PAH when they were registered were collected as the baseline data. A case-control study was conducted by taking the admitted SLE-non-PAH patients adjusted for age and gender in a 4:1 ratio during the same period as the controls. The associated variables were examined by binary multivariate logistic regression analysis to identify possible risk factors. A total of 111 RHC-confirmed SLE-PAH patients were enrolled, with the onset age of 34.6 ± 8.6 years old and the average SLE duration of 5 years. RHC revealed mPAP as 46.4 ± 11.4 mm Hg, CI as 2.7 ± 0.8 L/min × m^2^, and PVR as 10.5 ± 4.8 WU. 46% of patients were WHO Fc I–II. All patients were treated with immunosuppressive agents and 65% patients had PAH-targeted therapy. The case-control study had confirmed 2 independent risk factors previously published: pericardial effusion (OR = 21.290, *P* < 0.001) and anti-RNP antibody (OR = 12.399, *P* < 0.001). Meanwhile, 6 independent variables were discovered: baseline SLE duration (OR = 1.118, *P* = 0.007), interstitial lung disease (OR = 17.027, *P* < 0.001=, without acute rash (OR = 3.258, *P* = 0.019), anti-SSA antibody (OR = 4.836, *P* = 0.004), SLEDAI≤9 (OR = 26.426, *P* < 0.001), ESR≤20 mm/h (OR = 12.068, *P* < 0.001), and uric acid > 357 μmol/L (OR = 9.666, *P* < 0.001) to be associated with PAH in SLE patients.

The PUMCH study has shown that SLE patients complicated with PAH are usually earlier diagnosed and have less disease severity than patients without PAH. The immunosuppressive therapy rate and the PAH target therapy rate were high, which is consistent with reports from Western countries. This study has confirmed that pericardial effusion and positive anti-RNP antibody are risk factors for SLE-associated PAH. Long SLE disease duration, the presence of interstitial lung disease, without acute skin rash, positive anti-SSA antibody, low SLEDAI and ESR, and high uric acid levels are also associated with PAH in SLE patients.

## INTRODUCTION

Systemic lupus erythematosus (SLE) is one of the most complicated diseases among autoimmune diseases, with multiorgan involvement and diverse clinical manifestations, for example, lupus nephritis, lupus encephalopathy, and thrombocytopenia.^1^ Cardiac and pulmonary involvements, though not much attention has been paid, are severe complications with high morbidity and mortality rate,^2,3^ especially pulmonary arterial hypertension (PAH), which is the 3rd leading cause of death in Chinese SLE patients.^4^ Pulmonary hypertension (PH) consists of 5 groups and PAH is classified as Group I.^5–9^ CTD-associated PAH consisted 25%,^7^ following idiopathic PAH (IPAH) as the most common subtype in all Group I PH.^10,11^

CTD has a broad disease spectrum. With different prevalence and characteristics, almost every underlying disease might lead to PAH. Systemic sclerosis (SSc) and systemic lupus erythematosus (SLE) are the most common causes among the underlying CTD.^12–14^ For years, SSc-PAH has been recognized as a research model of CTD-PAH;^10^ however, SLE-PAH has been proven to have a lighter severity and better prognosis than SSc-PAH.^10,15^ In the literature, however, studies focused on SLE-PAH are few, and the study populations are not in a large scale, especially diagnosed by the gold standard RHC.^13,16,17^

SLE has a high frequency in Asian countries than Western world.^18^ Data from Chinese SLE Treatment and Research Group (CSTAR) has shown that the PAH prevalence in SLE patients is 3.8%,^19^ and a recent CTD-PAH study revealed that SLE was the most common underlying disease in Chinese CTD-PAH patients,^20^ which suggested that a large SLE-PAH patient population existed all over the country. PUMCH has started SLE-PAH treatment for 8 years. We analyzed data from this single-center RHC-based SLE-PAH study to present the baseline characteristics and at the mean time identify risk factors and associated variables of PAH in lupus. We believe the results of this study will provide valuable guidance to the prevention and management of this specific problem, as some of the clinical manifestations of SLE would provide helpful clues for earlier diagnosis of PAH, patients would benefit more from the progress achieved in PAH treatment recent years.^21,22^

## METHODS

### Patient Recruitment

This cross-sectional study was conducted based on 8-year's clinical experience in PUMCH. This study was approved by Medical Ethics Committee of PUMCH, and patients recruited all signed the written informed consent by themselves or their authorized guardians. After other CTDs were excluded, patients who met the 2012 Systemic Lupus International Collaborating Clinics (SLICC) classification criteria for SLE were enrolled.^23^ Patients’ information, including demographic data, clinical manifestations, laboratory test results, and treatment regimens were collected in a protocol-directed method by well-trained rheumatologists. In the meantime, we selected lupus patients from PUMCH who met the definition of PAH as below. The control group was selected in a 4:1 ratio by choosing admitted SLE-non-PAH patients during the same period in PUMCH adjusted with age and gender.

### PAH Definition

Initial diagnosis or screening of PAH was based on echocardiography (ECHO) when the systolic pulmonary arterial pressure (PASP) ≥ 40 mm Hg, which is a hemodynamic index estimated based on tricuspid regurgitant jet velocity (TRV).^24,25^ As right heart catheterization (RHC) is well accepted as the gold standard for PAH diagnosis, PAH is defined as mPAP ≥ 25 mm Hg, PAWP ≤ 15 mm Hg and PVR > 3 Woods by RHC^5^ in our study. All patients were further checked based on the 2012 SLICC SLE diagnosis criteria.^23^ The exclusion criteria included: ① PAWP > 15 mm Hg during RHC performance; ② total lung capacity (TLC) < 60% by pulmonary function test (PFT);^10^③ ventilation perfusion scintigraphy (V/Q) or computed tomographic pulmonary angiography (CTPA) showed possible thromboembolism; ④ overlapping CTDs. SLE-non-PAH patients in the control group were defined as PASP < 40 mm Hg by echocardiography.

### Data Collection

We used a uniform evaluation chart to collect patients’ information, including demographic data, SLE classification criteria, clinical manifestations, autoantibodies, laboratory results, and treatment regimens in both SLE-PAH and SLE-non-PAH groups. Meanwhile, we designed another form for PAH data collection, which contained PAH initial symptoms, diagnosis, evaluation, and therapy.

Demographic data includes gender, height, weight, age at SLE onset, age at SLE diagnosis, age at recruitment, past medical history, socioeconomic status, reproductive history, and family history of rheumatic diseases. For PAH patients, age at PAH onset and diagnosis were also recorded. Recruitment date was defined as the RHC date for SLE-PAH patients and was the admission date for SLE-non-PAH patients. Diagnosis date was defined as the initial clinical diagnosis date for SLE and abnormal ECHO date for PAH. SLE diagnostic criteria used in this study were SLICC 2012, and lupus disease activity was evaluated by SLEDAI (SLE disease activity index). Autoantibodies were routinely tested by Laboratory Department in PUMCH, positivity of antinuclear antibody (ANA), anti-dsDNA, anti-Sm, anti-SSA, anti-SSB, anti-RNP, anti-rRNP, anticardiolipin, anti-β2GPIantibody, and lupus anticoagulant were tested. Other laboratory results included complete blood count, liver function test, renal function panel, urine analysis, hs-CRP, ESR (erythrocyte sedimentation rate), serum complement, and immunoglobulin levels. For PAH patients, we also collected their BNP, NT-pro BNP, cardiac enzymes, uric acid levels as well as the indexes of RHC, ECHO, and PFT. For treatment regimens, steroid and immuno-suppressors were documented for the dosage, administration route, and the initiation date. Diuretics, digoxin, anti-platelet or anti-coagulation, calcium channel blocker (CCB), and PAH-targeted medicine were also recorded and analyzed.

### Statistical Analysis

Variables were described by counts or percentages using medians and ranges. For categorical data, chi-squared tests and Fisher's exact tests were used, whereas for quantitative data, independent *t* tests were used for comparison between case and control groups. *P* values < 0.05 were considered as statistically significant. Risk factor candidates with statistically significance were investigated by multivariate binary logistic regression analysis, and the results were presented using odds ratios and 95% confidence intervals. Data were analyzed by SPSS 19.0 (SPSS Inc, Chicago, IL).

## RESULTS

### Demographic Data

A total of 133 SLE patients underwent right heart catheterization, 12 with mPAP < 25 mm Hg, 3 with PAWP >15 mm Hg, 7 with positive V/Q or CTPA result, 0 with TLC < 60%. A total of 111 SLE-PAH patients were actually included into our research.

Female-to-male ratio was 108:3. Average age at lupus and PAH onset was 27.7 ± 9.1 and 32.9 ± 8.6 years old respectively. SLE duration at recruitment was 7.0 ± 6.3 years, PAH onset was found to be 5.2 (±6.0) years after the diagnosis of SLE, and diagnosis by RHC was made with a 1.8 (±2.0) years’ delay from onset.

### PAH Onset, Evaluation, and Treatment Regimen

We recorded the initial manifestations of PAH in SLE disease course. Totally 84.5% patients complained of short of breathlessness (SOB) at onset, and other common symptoms included fatigue (34.5%), cough (28.2%), palpitation (16.4%), peripheral edema (16.4%), chest pain (15.5%), syncope (14.5%), dyspnea (13.6%), and distension (5.5%).

For PAH evaluation, RHC revealed mPAP 46.4 ± 11.4 mm Hg, CI 2.7 ± 0.8 L/min×m^2^, and PVR 10.5 ± 4.8 WU. Apart from RHC, other meaningful indexes were right ventricular structure by ECHO, WHO Functional class (Fc), 6MWD, BNP, NT-pro BNP, and they were described in Table 1. Right ventricular diameter was 31.7 ± 6.8 mm in our SLE-PAH group. A total of 46% patients were in WHO Fc I–II; 6MWD was 423.2 ± 92.4 m on average. BNP and NT-pro BNP levels were 468.2 ± 981.7 ng/L and 1767.9 ± 2128.4 pg/mL, respectively.

**TABLE 1:**
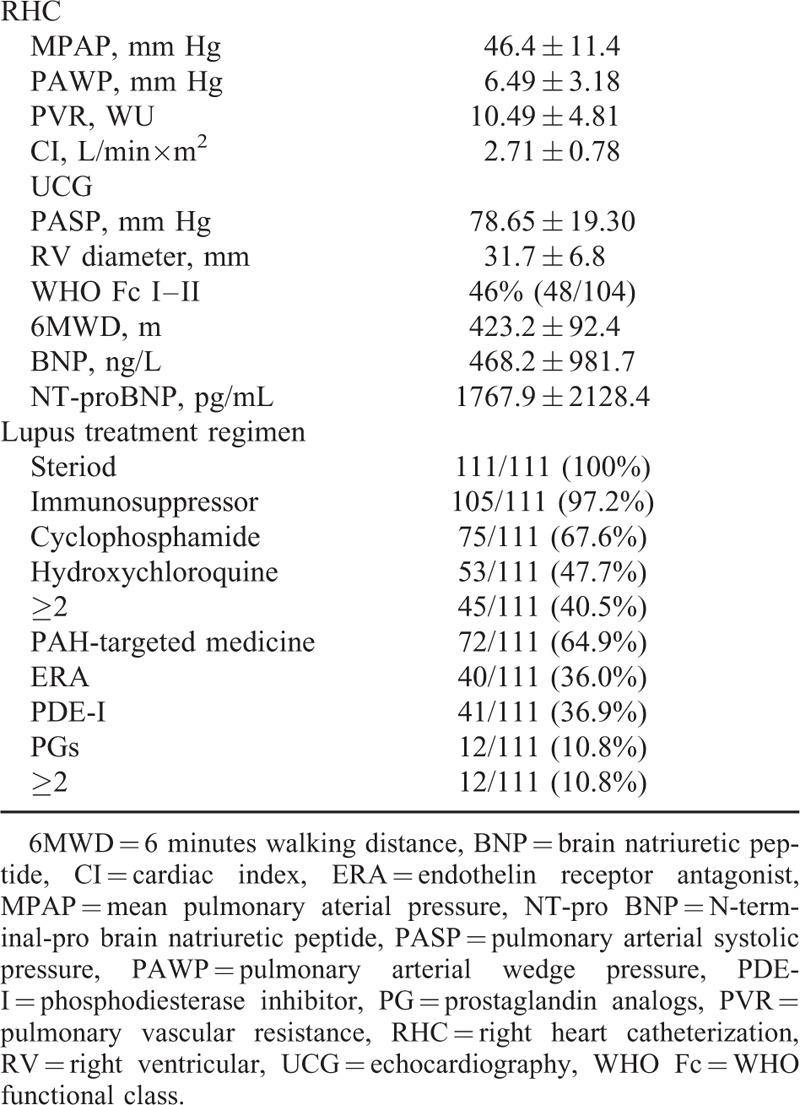
PAH Evaluation and Treatment Regimens of SLE-PAH Patients From PUMCH

Treatment for SLE-PAH consisted therapies for SLE and PAH. Immunosuppressive therapy (IST) was initiated in all patients, with steroid (100%) or immunosuppressors (97.2%). For immunosuppressors, 67.6% SLE-PAH patients were treated with cyclophosphamide, and 47.7% were treated with hydroxychloroquine. And 40.5% patients were treated with >2 immuno-suppressors simultaneously. As for PAH therapy, 64.9% SLE-PAH patients were treated with PAH-targeted medicine, and 10.8% were prescribed with >2 targeted medications. The percentages for ERA (endothelin receptor antagonist), PDE-I (phosphodiesterase inhibitor), and prostaglandin analogues (PGs) were 36.0%, 36.9%, and 10.8%, respectively.

### Lupus diagnosis and evaluation

A total of 444 simultaneously admitted SLE-non-PAH patients were selected as the control group and were adjusted for age and gender. Lupus duration at recruitment was 3.9 ± 4.6 versus 7.0 ± 6.3 years (*P* = 0.001), and interstitial lung disease (ILD) by high-resolution computed tomography (HRCT) (3.2% vs 29.7%, *P* < 0.001) showed significant statistical differences in SLE-PAH and SLE-non-PAH groups. Pericardial effusion proved by ECHO was in 52.5% SLE-PAH patients and in 10.6% SLE-non-PAH patients, *P* < 0.001.

For the accordance with each criteria of 2012SLICC in both case and control groups, the analysis showed that the occurrence rates of acute rash and lupus nephritis were lower in the SLE-PAH group (40.9% vs 52.7%, *P* = 0.033; 41.8% vs 55.4%, *P* = 0.014), whereas alopecia and serositis were higher (37.3% vs 26.4%, *P* = 0.025; 65.8% vs 21.2%, *P* < 0.001).

According to Table 2, positivity of anti-dsDNA, anti-Sm, anti-RNP, and anti-SSA antibody were significantly elevated in the case group than the controls.

**TABLE 2:**
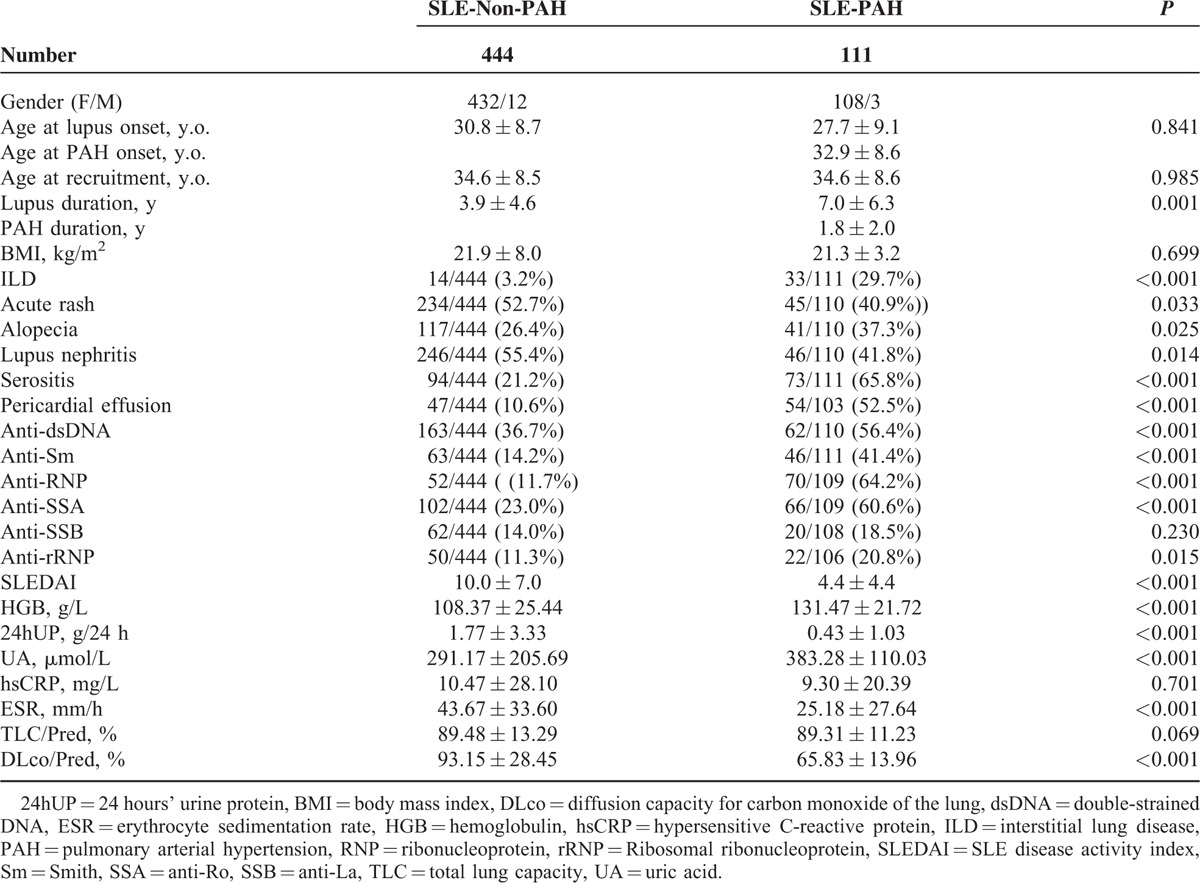
Baseline Demographic and Clinical Characteristics of SLE-PAH and Non-PAH Patients

When the lab results were compared, SLEDAI was found to be reduced in SLE-PAH patients (4.4 vs 10.0, *P* < 0.001). Other significantly decreased laboratory tests were: 24hUP (urine protein), ESR and DLco, whereas hemoglobulin and uric acid were elevated in SLE-PAH patients.

### Independent-associated Variables for PAH in SLE Patients

Multivariate binary logistic regression analysis of 16 statistically significant candidates was used to confirm independence. Associated variables included were as following: lupus duration, ILD, acute rash, lupus nephritis, alopecia, serositis, pericardial effusion, anti-dsDNA, anti-Sm, anti-SSA, anti-RNP, and anti-rRNP, SLEDAI≤9, ESR≤20 mm/h, hemoglobin>150 g/L, and uric acid>357 μmol/L. 24hUP were index of SLEDAI and DLco were in accordance with ILD; thus they were not included in further analysis.

Our case-control study discovered 9 independent associated variables for PAH in lupus patients, which were: baseline SLE duration (OR = 1.118, *P* = 0.007), interstitial lung disease (OR = 17.027, *P* < 0.001), without acute rash (OR = 3.258, *P* = 0.019), pericardial effusion (OR = 21.290, *P* < 0.001), anti-RNP antibody (OR = 12.399, *P* < 0.001), anti-SSA antibody (OR = 4.836, *P* = 0.004), SLEDAI≤9 (OR = 26.426, *P* < 0.001), ESR≤20 mm/h (OR = 12.068, *P* < 0.001), and uric acid>357 μmol/L (OR = 9.666, *P* < 0.001). Odds ratios, 95% confidential intervals, and *P* values for each associated variable were listed in Table 3.

**TABLE 3:**
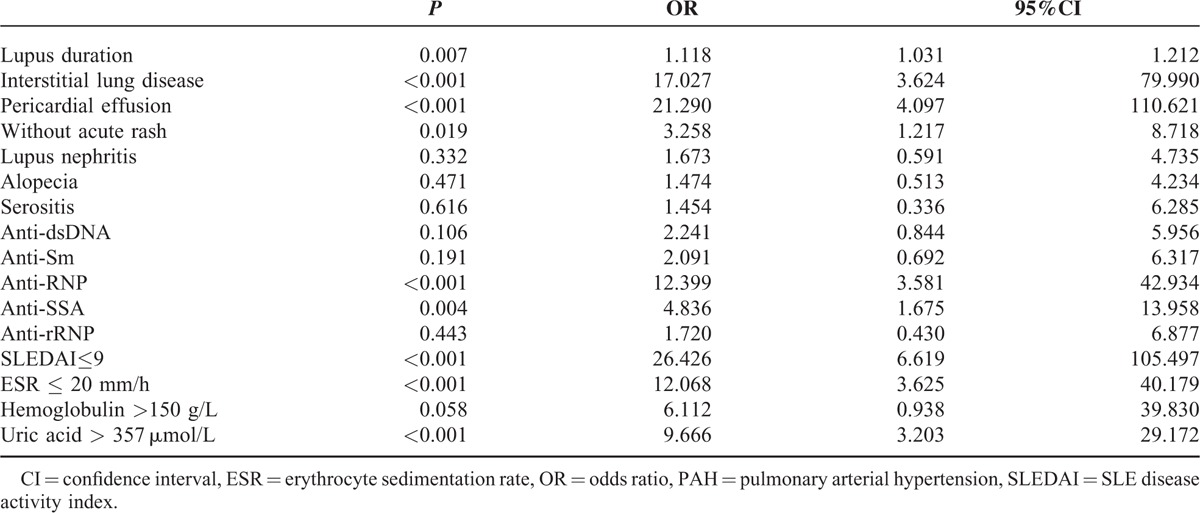
Multivariate Binary Logistic Regression Analysis of SLE-PAH-Associated Variates

## DISCUSSION

### Demographic Data

Demographic data showed female predominance. Age at recruitment was 34.6 ± 8.6 years old. Previous studies in Asian population reported the average age of 32–40.6,^26,27^ whereas in Western countries patients that was 42 to 45.5,^10,15^ which indicated an earlier onset age in Asian SLE-PAH patients. Difference between PAH onset and lupus onset was 5.19 ± 6.02 years, similar with 4.9 ± 3.7 reported in the literature.^13^ However, PAH might occur at any time during lupus, even as initial manifestation.^28,29^

PAH diagnosis was made 1.8 ± 2.0 years after the onset in PUMCH, whereas the delay was 3 years reported in the literature.^13^ Jing et al in 2011 reported that the delay was 2.9 ± 3.3 years in China.^11^ This decrease of diagnosis delay might be an indication of the improved awareness of PAH in CTD patients by rheumatologists.

### PAH Evaluation

In our study, mPAP was consistent with other Asian data (46.4 vs 42^27^ vs 48^26^ mm Hg) whereas lower than US and UK cohort (46.4 vs 46.6^10^ vs 48^15^ mm Hg), as showed in Table 4. Apart from mPAP, lower PVR, higher CI, together with an elevated percentage of WHO Fc I–II and elongated 6MWD, supported a better status of our patients.^10,11,15,26,27,30^ Considering a shorter diagnosis delay, PAH duration might result from different severities of disease. Jing et al reported an average Chinese CTD-PAH mPAP of 50 mm Hg^11^ and Zhang et al reported as 51.1 mm Hg,^20^ higher than reported by PUMCH. As their patients were consisted of SSc- and SLE-PAH, so this discrepancy might be explained by different severities between SSc- and SLE-PAH patients, which had been proven by several studies.^10,15,20^

**TABLE 4:**
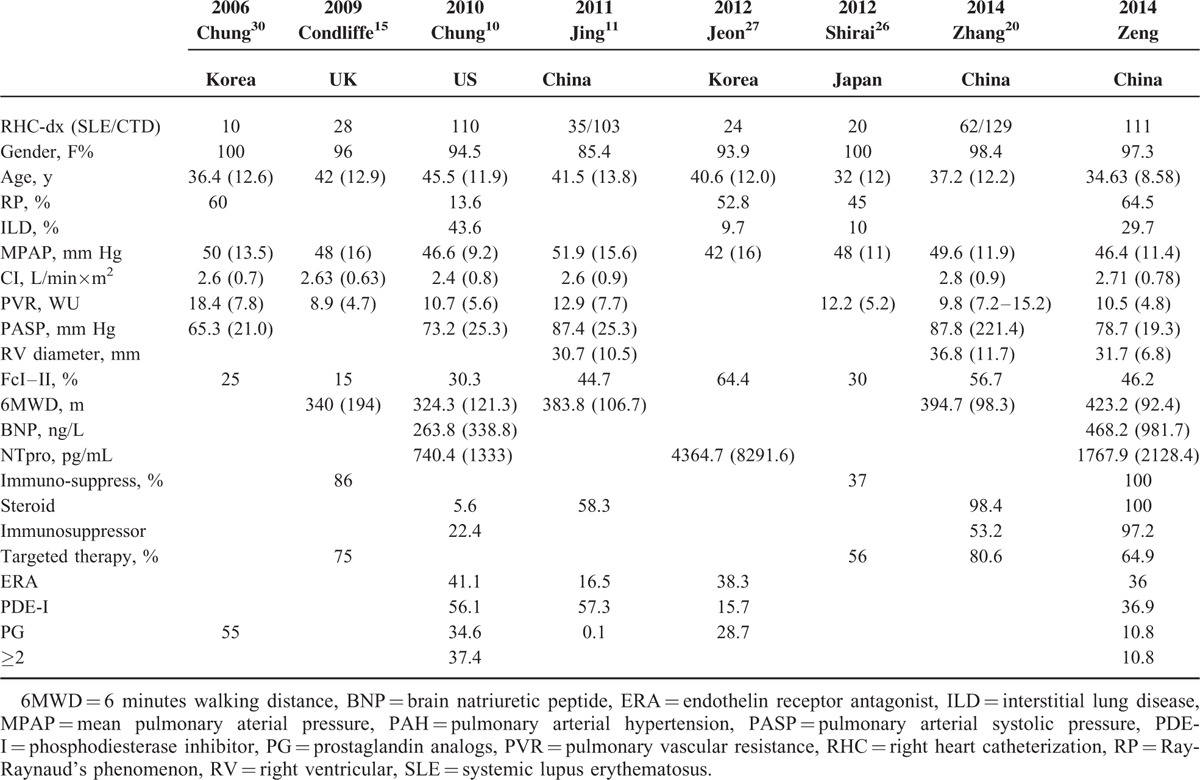
Literature Review and Comparison of Demographic, PAH Evaluation, and Treatment in SLE-PAH

### PAH Treatment Regimen

PAH treatment has been improved as targeted therapies are available. However, efficacy studies of these medications were mainly focused on idiopathic PAH, and few studies for CTD-PAH.^11,15,26,31^

Whether patients would benefit from immuno-suppressive therapy is another question to be answered.^32–35^ For patients in WHO Fc I/II or in Fc III with CI>3.1 L/min×m^2^, steroid and cyclophosphamide combination therapy were recommended.^34^ Clinical experience from the single center study has shown that good responders to this immune-suppressive regimen were in better status than nonresponders, which means, with better WHO Fc and RHC indexes,^33^ higher anti-dsDNA and SLEDAI.^34^

In PUMCH, SLE disease control and PAH treatment weighed equally as rheumatologists were the main participants during the treatment process. Patients all got immuno-suppressive therapy in our center, and PAH-targeted treatment percentage was also at the similar level as in developed countries. But the effectiveness of PAH-targeted and immuno-suppressive therapy on the prognosis needs further studies.

### Risk Factors and Associated Variable for PAH in Lupus

Previously proved risk factors in the literature included Raynaud's phenomenon, anti-phospholipid antibody, anti-RNP antibody, serositis, the presence of pericardial effusion, anti-endothelial cell antibody, endothelin, disease activity, and so on.^13,36–40^ Data based on CSTAR has shown pericardial effusion, serositis, and anti-RNP antibody are independent risk factors in ECHO-based SLE-PAH patients,^19^ and later analysis of auto-antibodies has found that there is relevance between anti-SSA antibody and PAH.^41^

Three mechanisms participate in PAH for Lupus patients. One mechanism is vasculopathy. This group mimics SSc-PAH, in which irreversible vascular pathological changes, especially proliferation of endothelial cells and vascular smooth muscle cells, gradually lead to PAH during long disease duration. Anti-RNP antibody and Raynaud's phenomenon (RP) were believed to be associated with this subtype,^14,19,34,42^ and RP was also proved as an indicator of disease severity.^36,38,42–44^ Percentage of RP in our study was 64.5%, in accordance with previous published data;^45^ however, we lack the data of the control group. Thus we could not prove it as a risk factor. Nevertheless, we could suggest that PAH screening should be performed for SLE patients with positive anti-RNP antibody, especially when associated with RP.

Long lupus duration was found as an independent associated variable in our study. By choosing the adjusted control group, we balanced the consequence of disease nature. As mentioned, the SSc-mimic group was more characterized with irreversible vasculopathy, it was reasonable that disease duration played a role. SLEDAI and ESR was lower in the PAH group, which was also a good supporting point, as long lupus duration would contribute to a more stable disease condition, especially with a high immuno-suppressive therapy rate. SLE was intended to be well controlled before the RHC procedure as well. Thus, risk for PAH would be increased as time passes, even though disease might be well controlled.

Interstitial lung disease would decrease effective gas exchange and led to a decreased DLco. ILD could cause Group 3 PH (due to chronic lung disease and/or hypoxia). TLC<60% suggest patient has severe ILD, then PH might be a result of chronic lung disease apart from underlying connective tissue disease. But it was not rare for ILD as comorbidity in CTD, especially when ILD was 29.7% in our study and 43.6% in REVEAL. We suggest that rheumatologists should be aware of PAH in lupus patients with ILD, even when TLC>60%.

PAH could also be vasculitis in lungs as part of systemic inflammatory response, it is believed to be the second mechanism, and the subgroup would benefit more from immuno-suppressive therapy.^14^ We proved that the presence of pericardial effusion was an independent risk factor, but it is hard to distinguish whether it was the result of high lupus activity or a cardiological pathophysiological change. Further stratified analysis might be helpful to provide meaningful answers.

A thrombotic mechanism was believed to play an important role in PAH, even though previous studies were controversial.^37,39,45^ The presence of antiphospholipid antibodies might suggest thrombosis in situ. But we were not able to prove any relevance between antiphospholipid antibodies and PAH, which needs more stratified analysis.

The association of anti-SSA antibody with SLE-PAH was first reported in a study from CSTAR.^41^ Our study was able to prove this association in clinical situation. We highly recommend a screen for PAH in lupus patients with secondary Sjogren's syndromes. Uric acid would increase in hypoxia condition and has been reported as an independent risk factor, risk stratification index, and prognostic factor for PAH.^7,46,47^ Another interesting finding was that acute rash seemed to be a protective factor for PAH in lupus patients. We tend to believe change of appearance would raise more attention for diagnosis and evaluation, but more exhaustive study was needed.

Our study has several limitations. First, as a tertiary center, SLE-PAH patients included in this study were mostly referred to PUMCH and most patients recruited were already treated at local hospitals; thus delay between PAH onset and RHC performance could not be avoided, so our patients cohort could hardly be considered as an inception cohort, which will affect the results in a certain extent. Second, a perspective cohort study on PAH-risk patients with SLE might be needed to validate risk factors and associated factors found in our cross-sectional study. Third, prognostic data in our center should be tracked to support the efficacy of immunosuppressive therapy in SLE-PAH patients and that would be our next work.
